# *H. pylori* infection and osteoporosis: a large-scale observational and mendelian randomization study

**DOI:** 10.1186/s12879-024-09196-1

**Published:** 2024-03-12

**Authors:** Ling Zhang, Daya Zhang, Ling Wei, Yan Zhou, Ximei Li, Runxiang Chen, Xiaodong Zhang, Shiju Chen, Feihu Bai

**Affiliations:** 1 Department of Hospital infection management, LinYi people’s Hospital, LinYi, Shandong Province China; 2 Graduate School of Hainan Medical University, Haikou, China; 3 Department of Gastroenterology, The Second Affiliated Hospital of Hainan Medical University, Yehai Avenue, #368, Longhua District, Haikou, Hainan Province China; 4 The Gastroenterology Clinical Medical Center of Hainan Province, Haikou, China; 5 The Third School of Clinical Medicine, Ningxia Medical University, Yinchuan, China

**Keywords:** *Helicobacter pylori*, Osteoporosis, Serum albumin, High-density lipoprotein, Fasting blood glucose, Vitamin D, Mendelian randomization

## Abstract

**Purpose:**

There is controversy concerning the relationship between Helicobacter pylori (*H. pylori*) infection and osteoporosis. This study is to examine the causal relationship between *H. pylori* infection and osteoporosis and to analyze the potential mechanism underlying the relationship.

**Methods:**

The clinical data of *H. pylori* infection and bone mineral density from patients or physical examiner with good general condition in our hospital between September 2019 and September 2020 were retrospectively collected. The relationship between *H. pylori* infection and osteoporosis was compared and analyzed, using logistic regression to examine the potential mechanism underlying the association. To investigate the causal effects of *H. pylori* infection and osteoporosis, we conducted a two-sample bidirectional Mendelian randomization (MR) analysis.

**Results:**

A total of 470 patients were positive for *H. pylori*, with a detection rate of 52.22%. It was found that age, SBP, FPG, DBP, ALB, LDL-C, hs-CRP, and OC were positively correlated with osteoporosis, while negative correlations were observed with BMI, LYM, ALB, TP, TG, HDL-C, SCr, UA, and VitD. After stratified analysis of sex and age, it was found that there was a significant correlation between *H. pylori* infection and osteoporosis. The levels of SBP, ALP, FPG, LDL-C, hs-CRP, and OC in both *H. pylori-*positive group and osteoporosis group were higher than those in the *H. pylori*-negative group while the levels of BMI, ALB, TP, HDL-C, SCr, UA, and VitD in the positive group were significantly lower than those in the negative group. Logistic regression analyses with gender and age showed that ALB, FPG, HDL-C, and VitD were common risk factors for osteoporosis and *H. pylori* infection. In the MR analysis, the IVW results found a positive effect of *H. pylori* infection on osteoporosis (OR = 1.0017, 95% CI: 1.0002–1.0033, *P* = 0.0217). Regarding the reverse direction analysis, there was insufficient evidence to prove the causal effects of osteoporosis on *H. pylori* infection.

**Conclusion:**

Our study provides evidence for causal effects of *H. pylori* infection on osteoporosis. *H. pylori* may affect osteoporosis through serum albumin, high-density lipoprotein, fasting blood glucose and vitamin D.

**Supplementary Information:**

The online version contains supplementary material available at 10.1186/s12879-024-09196-1.

## Background

*Helicobacter pylori (H. pylori)* is a Gram-negative bacillus that adversely affects digestive health [1]. It is a micro-anaerobe and can colonize the surfaces of gastric mucosal epithelial cells for extended times through its ability to hydrolyze urea to produce ammonia and CO_2_ to neutralize gastric acid [1]. *H. pylori* infection mainly occurs through mouth-to-mouth and feces-mouth transmission and is widely found in human populations of different ethnicities and regions [2]. Epidemiological studies have shown that nearly half of the global population is infected with *H. pylori* [3]. The association between *H. pylori* infection and gastrointestinal disease was first proposed in 1989 [4]. In 1994, the World Health Organization identified *H. pylori* as a class I carcinogen [5]. Since then, *H. pylori* infection has been demonstrated to be closely linked to a variety of parenteral diseases.

Osteoporosis is a common bone disease characterized by decreased bone mass and degeneration of the bone tissue microstructure, leading to increased bone brittleness and a high risk of fracture [6]. Osteoporosis has been linked to a variety of causes [7]. It has been found that *H. pylori* influences bone metabolism by affecting inflammatory reactions, oxidative stress, blood lipid metabolism, and insulin resistance, all of which have been shown to promote the occurrence and development of osteoporosis [8]. However, a study by Kakehasi [9] found that *H. pylori* infection did not adversely influence bone mineral density and was not a risk factor for reduced bone density in healthy women. There is thus controversy concerning the relationship between *H. pylori* infection and osteoporosis, and the underlying causes need to be further explored.

Mendelian randomization (MR) is a new method for genetic variation to assess causality between exposure and outcome [10–11]. It can avoid confounders and infer causality because alleles for exposure genetic variants are randomly assigned [10–11]. However, there is a lack of evidence on a causal relationship between *H. pylori* infection and osteoporosis.

In this study, we first examined the observational association between *H. pylori* infection and osteoporosis in a large cohort from our hospital’s database. We then performed a bidirectional MR analysis using genome-wide association study (GWAS) data to examine the causal relationship between *H. pylori* infection and osteoporosis. This analysis may assist in the clarification of the role of *H. pylori* in the pathogenesis of osteoporosis and suggest new strategies for prevention and treatment.

## Methods

### Study design

Firstly, we conducted a retrospective analysis to explore the association between *H. pylori* infection and osteoporosis. Secondly, we used bidirectional MR analysis to investigate the causal effect between *H. pylori* infection and osteoporosis. This study was approved by the Ethics Committee of our Hospital (Reference No. LW20221025). The need to obtain informed consent was waived because the data used for MR analysis were anonymous and readily available to the public. Furthermore, this study was conducted in accordance with the 1964 Declaration of Helsinki and its subsequent amendments [12]. In addition, the study is enhancing the reporting of observational epidemiological studies using the Mendelian randomization (STROBE-MR) guidelines [13].

### Observational analysis: data source

#### Patients

Clinical data on *H. pylori* infection and bone mineral density in patients or physical Examiner with good general condition admitted to Ningxia Hui Autonomous People’s Hospital between September 2019 and September 2020 were retrospectively collected. Exclusion criteria: (1) Use of antibiotics, acid suppressants, bismuth, and other drugs that interfere with the detection of H. pylori or drugs that affect bone metabolism, such as anticonvulsants, glucocorticoids, cyclosporine, calcium-vitamin D-Fluoride bisphosphonates - calcitonin - HRT and a total vegetarian diet four weeks before testing; (2) Fasting or fasting for fewer than three hours before examination; (3) History of resection or transplantation of digestive organs; (4) Bedridden for more than three months. (5) Pregnant or lactating women; (6) Presence of malignant tumors, thyroid disease, history of chronic inflammatory diseases (RA, SLE, AS, IBD), chronic liver disease, chronic kidney disease, Cushing’s disease, steroid osteoporosis, or kidney disease as well as malabsorption caused by osteodystrophy or other metabolic and gastrointestinal diseases; (7) Incomplete clinical data. The presence of H. pylori and the degree of infection were diagnosed by the 14 C breath test. The bone mineral density of lumbar vertebrae 2–4 was measured by dual-energy X-ray absorptiometry. The diagnosis and degree of osteoporosis were assessed according to the guidelines for diagnosis and treatment of Primary Osteoporosis (2017).

#### Observation indicators

General data: sex (male, female), age, body mass index (BMI), systolic blood pressure (SBP), diastolic blood pressure (DBP); routine blood examinations: white blood cell count (WBC), neutrophil percentage(NEU), lymphocyte percentage (LYM); Biochemical tests: calcium (Ca), phosphorus (P), magnesium (Mg), alkaline phosphatase (ALP), albumin (ALB), total protein (TP), fasting blood glucose (FBG), total cholesterol (TC), triglyceride (TG), high-density lipoprotein (HDL-C), low-density lipoprotein (LDL-C), creatinine (SCr), uric acid (UA), and hypersensitive C-reactive protein (hs-CRP); Bone metabolism: vitamin D (VitD), parathyroid hormone(PTH), osteocalcin (OC); bone mineral density: BMD value, T-value, Z-value; ^14^C breath test: DPM value.

#### Detection of *H. pylori*

Patients were instructed to swallow one ^14^C-urea capsule with water on an empty stomach. After waiting for about 15 min, the patient was instructed to exhale through the mouth for approximately 2–5 min until the color of the indicator window on the expiratory card changed from blue to white or a small amount of blue remains, or the timing reaches 5 min. After completion of the information on the breath card, the seal on the test window was removed and the card was inserted into the tester. The results were complete in approximately 4 min and were calculated according to the disintegrations per minute, with results < 100 dpm/mmol CO_2_ indicating a negative result and those ≥ 100 dpm/mmol CO_2_ being positive.

#### Determination of bone mineral density

The bone mineral density of lumbar vertebrae 2–4 was measured by a professional radiologist using dual-energy X-ray absorptiometry. Osteoporosis was diagnosed according to the guidelines for diagnosis and treatment of Primary Osteoporosis (2017) [14] as follows: in males ≥ 50 years old and postmenopausal females, a T-value≥-1.0 indicates low bone mass, -2.5 < T <-1.0 indicates osteoporosis, a T-value ≤-2.5 represents severe osteoporosis, while T-values ≤-2.5 + are indicative of brittle fracture. In children, males < 50 years old, and premenopausal females, a T-value ≤-2.0 indicates low bone mass.

#### Measurement of serum indices

Fasting venous blood was collected in the early morning. The serum was separated by centrifugation and the biochemical indices were measured by immune transmission turbidimetry (ADVIA2400, Siemens, Germany). The normal reference values of the biochemical indices are as follows: TC, 3.35‒6.46 mmol/L; TG, 0.48‒2.00 mmol/L; HDL-C, 0.71‒1.68 mmol/L; LDL-C, 1.9‒3.8 mmol/L; FBG, 3.8‒6.4 mmol/L; Ca, 2.11‒2.52 mmol/L; P, 0.85‒1.51 mmol/L; Mg, 0.75‒1.02 mmol/L; ALP, 50‒135 U/L; ALB, 40‒55 g/L; TP, 65‒85 g/L; creatinine, 41‒81 umol/L; UA, 140‒420 umol/L; hypersensitive CRP, 0‒6 mg/L. Routine blood measurements were made on a Sysmex XE-5000 automatic blood cell analyzer. The normal reference values are 3.5‒9.5 × 10^9^/L for white blood cells, 40‒75% for neutrophils, and 20‒50% for lymphocytes. The normal values for bone metabolism were 25-hydroxyvitamin D, 30‒100 mg/ml; parathyroid hormone, 12.4 ‒76.8 pg/ml, and osteocalcin, 11‒48 ng/ml.

### MR analysis: data source

GWAS summary data for *H. pylori* infections were obtained from publicly available data in the European Bioinformatics Institute (EBI) database at https://gwas.mrcieu.ac.uk/datasets/ieu-b-4905/, which includes 1058 European cases and 3625 European control cases. GWAS osteoporosis summary data were obtained from public data in the MRC Integrated Epidemiology Unit (IEU) database at https://gwas.mrcieu.ac.uk/datasets/ukb-a-87/, which includes 5266 European cases and 331,893 European controls. Each GWAS was approved by the corresponding ethics committee. Details of the GWAS data included in this study are shown in Supplementary Table 1.

### Statistical analysis

SPSS 19.0 statistical software (IBM Corp., Armonk, NY, USA) was used for statistical analysis. The number of cases used in counting data was expressed by number (N) and percentage (%), and inter-group comparisons were made using χ^2^ tests. Normally distributed measurement data were expressed as means ± standard deviation (x ± s), and non-normally distributed data by quartile M (P_25_ ∼ P_75_). The Mann-Whitney U test was used for comparison between the two groups, and the F test or Kruskal-Wallis H test was used for comparison between groups. Binary logistic regression analysis was used for multivariate analyses. *P*-values < 0.05 were considered statistically significant. Spearman’s rank correlation test was used to measure associations, expressed by -1‒1 with higher values indicating a stronger association and the positive and negative signs used to represent positive and negative correlations, respectively. The Mantel-Haenszel hierarchical chi-square test was used to eliminate the influence of confounding factors on the results.

Bidirectional MR methods were used to estimate the causal relationship between *H. pylori* infection and osteoporosis. Relevant SNPs were selected when genome-wide significance was less than *p* < 5 × 10^− 8^. SNPs were excluded when linkage disequilibrium (LD) was detected (R^2^ > 0.001 or clump spacing < 10,000 kb). Five methods were used to test for causality, including inverse variance weighting (IVW), MR Egger, weighted mode, weighted median, and simple model, with IVW being the primary method [15]. Random effects IVW was used to assess the genetic prediction between them. MR Egger method was used to detect their horizontal pleiotropy. Outlier SNPs were detected by the MR-PRESSO package and then removed. Cochran’s Q statistic was applied to test the heterogeneity of individual SNPs in IVW and MR Egger tests. We also performed sensitivity analysis by removing individual SNPs one by one. All data were analyzed using the “two-sample MR” package in R language.

## Results

### Status of *H. pylori* infection in subjects

Among the 900 subjects included in the study, 470 were found to be *H. pylori*-positive, with a positive rate of 52.22%. Among them, 40.06% of males (125/312) and 58.67% of females (345/588) were positive. The *H. pylori* infection rate was significantly higher in females than in males (χ^2^ = 27.718 *P* < 0.001). *H. pylori*-infected patients tended to be older (60.46 ± 16.63) than uninfected patients (51.82 ± 17.77) years in Table 1.

**Table 1:** *H. pylori* infection in the subjects [n(%), x ®±s]

Grouping	N	Sex		Age (years)
		Male	Female	
*H. pylori* positive group	470(52.22)	125(40.06)	345(58.67)	60.46 ± 16.63
*H. pylori* negative group	430(47.78)	187(59.94)	243(41.33)	51.82 ± 17.77
χ^2^/t		27.718		7.53
*P*		<0.001		<0.001

### Comparison of osteoporosis indices

It was found that the incidence of osteoporosis was higher in females than in males (44.05% vs. 15.38%), with a statistically significant difference (χ^2^ = 76.579, *P* < 0.001). The rate of *H. pylori* infection was significantly greater in the osteoporosis group than in either the low bone-mass or normal bone-mass groups (53.82% vs. 33.62% vs. 12.55%; χ^2^ = 237.905, *P* < 0.001). Age, SBP, FPG, DBP, ALB, LDL-C, hs-CRP, and OC were positively correlated with osteoporosis, while BMI, LYM, ALB, TP, TG, HDL-C, SCr, UA, and VitD were negatively correlated with osteoporosis. ALB had the strongest correlation with osteoporosis (r_s_=-0.4), while LYM had the weakest correlation with osteoporosis (r_s_=-0.067) in Table 2.

**Table 2:** Analysis of osteoporosis indices [n(%), x ®±s, M(P25,P75)]

Project	Normal bone mass group(298)	Low bone mass group(295)	Osteoporosis group(307)	F/χ^2^ /H	r_s_	*P*
Sex(Male/Female)	141(45.19)/157(26.70)	123(39.42)/172(29.25)	48(15.38)/259(44.05)	76.579	-	<0.001
Age (years)	48.44 ± 17.48	55.68 ± 16.28	64.62 ± 15.49	73.732	0.374	<0.001
SBP(mmHg)	121(112,133)	128(117,140)	130(120,140)	29.828	0.181	<0.001
*H. pylori*(positive/Negative)	59(12.55)/239(55.58)	158(33.62)/137(31.86)	253(53.83)/54(12.56)	237.905	-	<0.001
FPG(mmol/L)	4.99(4.61,5.44)	4.98(4.65,5.45)	5.08(4.71,6.07)	7.598	0.081	0.022
DBP(mmHg)	75(68,83)	79(70,86)	78(70,87)	8.448	0.086	0.015
BMI(kg/m ^2^)	24.10(21.50,26.67)	23.90(21.70,26.20)	22.00(19.90,25.30)	45.779	-0.202	<0.001
WBC(*10^9^/L)	5.86(4.86,7.10)	5.72(4.79,6.84)	5.75(4.69,6.82)	1.896	-	0.387
NEU (%)	57.05(51.98,61.83)	56.80(50.50,62.80)	56.90(50.00,65.40)	1.665	-	0.435
LYM (%)	33.80(28.60,39.03)	34.20(28.60,39.60)	32.70(23.70,39.60)	6.356	-0.067	0.042
CA(mmol/L)	2.29(2.19,2.40)	2.26(2.19,2.37)	2.27(2.20,2.37)	0.437	-	0.804
P(mmol/L)	1.06(0.92,1.19)	1.11(0.99,1.19)	1.09(1.01,1.22)	2.427	-	0.297
MG(mmol/L)	0.89(0.84,0.96)	0.90(0.85,0.95)	0.91(0.84,0.97)	0.536	-	0.765
ALP(U/L)	64.90(52.00,84.00)	72.00(54.00,90.90)	74.00(58.50,95.00)	6.891	0.126	0.032
ALB(g/L)	49.00(43.00,52.80)	44.90(38.90,50.00)	39.50(36.50,44.80)	144.398	-0.400	<0.001
TP(g/L)	72.51(67.03,77.12)	69.45(64.99,74.30)	65.18(59.90,70.30)	116.203	-0.356	<0.001
TC(mmol/L)	4.43(3.79,5.03)	4.45(3.83,5.14)	4.46(3.78,5.22)	0.859	-	0.651
TG(mmol/L)	1.35(0.90,2.20)	1.49(1.05,2.12)	1.22(0.93,1.73)	14.541	-0.073	0.001
HDL-C(mmol/L)	1.35(1.11,1.63)	1.25(1.04,1.44)	1.17(0.99,1.39)	35.117	-0.195	<0.001
LDL-C(mmol/L)	2.11(1.56,2.57)	2.35(1.79,2.92)	2.31(1.91,2.92)	26.001	0.152	<0.001
SCr(umol/L)	62.98(53.58,73.32)	62.98(52.64,73.00)	59.00(51.00,69.00)	9.404	-0.093	0.009
UA(umol/L)	315.13(267.93,396.80)	295.85(248.00,359.87)	253.00(212.00,307.00)	95.513	-0.319	<0.001
hs-CRP(mg/L)	1.70(0.57,5.33)	2.30(0.80,9.80)	3.40(1.33,11.45)	12.403	0.173	0.002
VitD(ng/ml)	19.88(35.36,12.75)	13.87(8.81,19.67)	12.62(7.46,18.99)	26.351	-0.230	<0.001
PTH(pg/ml)	54.60(43.18,78.03)	52.20(40.30,77.10)	48.80(35.55,72.00)	4.515	-	0.105
OC(ng/ml)	13.81(9.37,19.13)	16.76(12.33,24.97)	18.73(13.57,26.27)	16.329	0.184	<0.001

BMI: body mass index; SBP: systolic blood pressure;DBP: diastolic blood pressure; WBC:white blood cell; NEU:neutrophil percentage;LYM: lymphocyte percentage; CA:calcium;P:phosphorus; ALP: alkaline phosphatase; ALB: albumin; FPG:fasting plasma glucose; TP:Total protein;SCr:serum creatinine;UA:uric acid;TC: total cholesterol;TG:triglyceride; HDL-C: high-density lipoprotein; LDL-C: low-density lipoprotein;CRP:C-reactive protein; VitD:vitamin D; PTH:parathormone;OC: osteocalcin

### Correlation between *H. pylori* infection and osteoporosis: M- H chi-square test

After adjustment for sex by the Mantel-Haenszel hierarchical chi-square test, it was found that there was still a significant correlation between *H. pylori* infection and osteoporosis (*P* < 0.001). The OR_M−H_ value and 95%CI were 0.451 (0.300-0.679). After adjustment for age, the OR_M−H_ and 95%CI values for *H. pylori* infection and osteoporosis were 0.232 (0.160–0.335) with a significant correlation (*P* < 0.001) in Table 3.

**Table 3:** Correlation between *H. pylori* infection and osteoporosis shown by the M-H chi-square test

Project	χ^2^	OR_M−H_	95%CI	*P*
Sex				
Male	32.321	0.246	0.150 ∼ 0.404	<0.001
Female	9.397	1.355	1.281 ∼ 1.434	0.002
*H. pylori* infection after sex adjustment	12.861	0.451	0.300 ∼ 0.679	<0.001
Age				
<60	74.606	0.179	0.119 ∼ 0.269	<0.001
≥ 60	4.081	1.318	1.241 ∼ 1.399	0.043
*H. pylori* infection after age adjustment	59.264	0.232	0.160 ∼ 0.335	<0.001

### Comparison between *H. pylori* infection and osteoporosis risk indices

A comparison of the two groups showed that the levels of SBP, ALP, FPG, LDL-C, hs-CRP, and OC in the *H. pylori*-positive group were significantly higher than those in the uninfected group. Furthermore, the levels of BMI, ALB, TP, HDL-C, SCr, UA, and VitD in the *H. pylori*-positive group were significantly lower than those in the *H. pylori*-negative group. There were no significant differences in DBP, LYM, and TG between the two groups (Table 4).

**Table 4:** Comparison of risk indices between *H. pylori* infection and osteoporosis [M(P25,P75)]

Project	H. pylori -positive group	H. pylori -negative group	Z	*P*
SBP(mmHg)	128(120,140)	123(114,136)	3.422	0.001
DBP(mmHg)	78(70,86)	77(69,83)	1.742	0.081
BMI(kg/m ^2^)	22.89(20.57,25.97)	23.60(21.40,26.31)	2.587	0.010
LYM (%)	33.05(26.18,39.60)	34.25(28.58,39.13)	1.532	0.126
ALP(U/L)	73.90(58.00,90.23)	65.80(52.00,86.50)	3.364	0.001
ALB(g/L)	40.80(37.40,47.30)	47.30(39.98,52.30)	8.438	<0.001
TP(g/L)	67.56(61.90,72.96)	70.91(65.38,76.08)	6.042	<0.001
FPG(mmol/L)	5.10(4.72,6.21)	4.91(4.57,5.34)	5.618	<0.001
TG(mmol/L)	1.30(0.97,1.88)	1.38(0.94,2.14)	1.005	0.315
HDL-C(mmol/L)	1.17(1.00,1.41)	1.32(1.10,1.59)	6.129	<0.001
LDL-C(mmol/L)	2.30(1.87,2.92)	2.18(1.63,2.64)	3.989	<0.001
SCr(umol/L)	60.58(51.70,71.00)	62.98(53.58,73.32)	2.006	0.045
UA(umol/L)	272.29(230.65,335.16)	305.28(251.00,375.88)	4.381	<0.001
hs-CRP(mg/L)	3.40(1.29,11.00)	1.70(0.70,6.43)	5.040	<0.001
VitD(ng/ml)	12.08(7.35,15.48)	23.28(14.59,35.36)	15.450	<0.001
OC(ng/ml)	17.72(13.12,26.20)	15.00(10.60,23.12)	4.629	<0.001

BMI: body mass index; SBP: systolic blood pressure;DBP: diastolic blood pressure; LYM: lymphocyte percentage; ALP: alkaline phosphatase; ALB: albumin; FPG:fasting plasma glucose; TP:Total protein;SCr:serum creatinine;UA:uric acid;TG:triglyceride; HDL-C: high-density lipoprotein; LDL-C: low-density lipoprotein;CRP:C-reactive protein; VitD:vitamin D; OC: osteocalcin

### Logistic regression analysis of common related factors between osteoporosis and ***H. pylori*** infection

Logistic regression analyses with gender and age showed that ALB, FPG, HDL-C, and VitD were common risk factors for osteoporosis and *H. pylori* infection (Table 5).

**Table 5:** Logistic regression analysis of common risk factors between osteoporosis and *H. pylori* infection

Variable	B	SE	Wald	*P*	OR	95%CI
ALB	-0.147	0.071	4.332	0.037	0.863	0.751 ∼ 0.991
FPG	1.284	0.140	83.907	<0.001	3.610	2.743 ∼ 4.751
HDL-C	-0.011	0.005	3.973	0.046	0.990	0.979 ∼ 1.000
VitD	-0.089	0.020	19.209	<0.001	0.914	0.879 ∼ 0.952

ALB: albumin; FPG:fasting plasma glucose; HDL-C: high-density lipoprotein; VitD:vitamin D

### Genetical prediction of *H. pylori* infection on osteoporosis

**Fig. 1:**
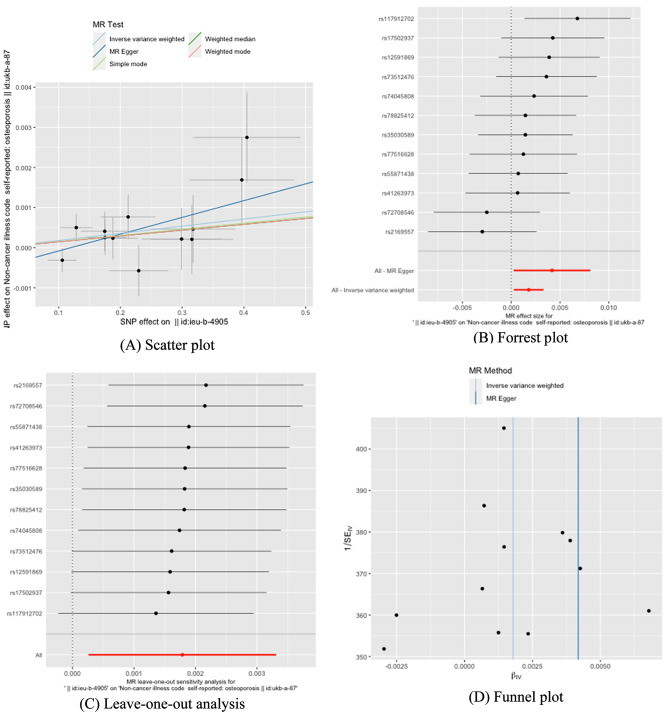
The causal effects of H. pylori infection on osteoporosis in different MR methods

Twelve SNPs were chosen as IVs for *H. pylori* infection (Supplementary Table 2). The causal association of *H. pylori* infection variants with osteoporosis was shown in Table 6; Fig. 1. The IVW results found a positive effect of *H. pylori* infection on osteoporosis (OR = 1.0017, 95% CI: 1.0002–1.0033, *P* = 0.0217). The Cochran’s Q statistic of MREgger (*P* = 0.518) and IVW methods (*P* = 0.457) indicated no significant heterogeneity between IVs. No horizontal pleiotropy was detected in MR–Egger intercept (*P* = 0.222). In the leave-one out sensitive analyses, we found rs117912702 strongly drove the overall effect of *H. pylori* infection on osteoporosis (Fig. 1C). Furthermore, the MR-PRESSO test (*P* = 0.2229) and IVW Radial (Supplementary Fig. 1) did not identify any potential SNP outliers.

**Table 6:** Causal effect of *H. pylori* infection on osteoporosis using genetic variant randomization in different MR methods

Exposure	Outcome	Method	No. of SNP	OR (95% CI)	95% CI	*P*
*H. pylori* infection	Osteoporosis	IVW	12	1.0017	1.0002–1.0033	0.0217
		MR Egger	12	1.0041	1.0002–1.0081	0.0631
		Weighted median	12	1.0014	0.9993–1.0035	0.1746
		Simple mode	12	1.0015	0.9981–1.0049	0.4066
		Weighted mode	12	1.0014	0.9981–1.0047	0.4238
MR Egger: Cochran’s Q = 9.144, *P* = 0.518						
IVW: Cochran’s Q = 10.833, *P* = 0.457						
MR-Egger intercept=−0.0005, *P* = 0.2229						
MR-PRESSO global test,*P* = 0.2229						

No outlier was observed in the MR-PRESSO analysis in MR analysis in *H. pylori* infection and osteoporosis. CI, confidence interval; MR, Mendelian randomization; IVW, inverse-variance weighted

### Reverse mendelian randomization analysis

Twenty-nine SNPs without linkage were chosen as the IVs for osteoporosis (Supplementary Table 3). Our study found no causal effect of osteoporosis on *H. pylori* infection by the IVW method (*P* = 0.797) (Table 7).

**Table 7:** Causal effect of osteoporosis on *H. pylori* infection using genetic variant randomization in different MR methods

Exposure	Outcome	Method	No. of SNP	OR (95% CI)	95% CI	*P*
Osteoporosis	*H. pylori* infection	IVW	29	/	/	0.7978
		MR Egger	29	/	/	0.0418
		Weighted median	29	/	/	0.5780
		Simple mode	29	/	/	0.3839
		Weighted mode	29	/	/	0.5019

CI, confidence interval; MR, Mendelian randomization; IVW, inverse-variance weighted

## Discussion

This study demonstrated that *H. pylori* infection were associated with an increased risk of developing osteoporosis. Further bidirectional MR analysis first revealed a significantly and causally link between *H. pylori* infection and osteoporosis.

*H. pylori* infection is low in infants and young children, with the infection rate increasing gradually with age and the frequency of social activities, which is in line with the characteristics of oral-to-oral transmission [16]. In this study, the average age of patients with *H. pylori* infection was 60.46 years, which was higher than that of 51.82 years seen in the *H. pylori*-negative group, which is consistent with the observation that the infection rate increases with age. Thus, the infection rate exceeded the *H*. *pylori*-negative rate after the age of 50 years. This age group also has a high incidence of other diseases. Although *H. pylori* are unable to enter the circulation, inflammatory mediators can be released into the blood resulting in a systemic inflammatory response and potentially leading to a variety of systemic diseases [17].

The etiology and pathogenesis of osteoporosis are related to many factors, some of which are genetic and others environmental, and are closely related to insulin resistance, damage caused by oxygen free radicals, and inflammation [18]. Some researchers have proposed that *H. pylori* may target the epiphysis and may affect bone growth by disrupting the osteoclast-osteoblast balance, leading to the destruction of osteoblasts during bone formation and eventually to osteoporosis [19]. A large cohort study found that people with *H. pylori* infection were 1.23 times more likely to develop osteoporosis than uninfected people and that there was a significant correlation between *H. pylori* infection and bone mass loss [20]. A meta-analysis including 9655 participants concluded that *H. pylori* infection increased the prevalence of osteoporosis (OR:1.39, 95%CI:1.13 ∼ 1.71) [21], although another meta-analysis did not find that *H. pylori* infection was a risk factor for osteoporosis (OR:1.49,95%:0.88 ∼ 2.55) [22]. Thus, the relationship between *H. pylori* infection and osteoporosis is still controversial. It has been suggested that *H. pylori* infection may cause chronic systemic inflammation and induce endocrine dysfunction, resulting in abnormal lipid metabolism and blood glucose levels [23]. In terms of the mechanisms by which *H. pylori* infection affects the pathogenesis of osteoporosis, the effect of *H. pylori* infection on local and systemic inflammatory responses is considered important, and *H. pylori* infection may indirectly diminish bone conversion. *H. pylori* infection causes chronic gastritis and induces an inflammatory response that increases the pro-inflammatory factors interleukins (IL-1β, IL-6, IL-8, IL-17), tumour necrosis factor α (TNF-α), interferon γ (IFN-γ), and C-reactive protein (CRP). In particular, virulent strains of *H. pylori* infection (e.g., CagA-positive (cytotoxin-associated gene A-positive) cause a stronger inflammatory response in the gastric mucosa and throughout the body [24]. Chronic *H. pylori* infection can predispose people to bone loss and osteoporosis, as bone cells are sensitive to pro-inflammatory cytokines [25]. Our study found that *H. pylori* may affect osteoporosis through serum albumin, HDL, fasting blood glucose and vitamin D, Which may be involved in the link that H. pylori infection stimulates local and systemic inflammatory factors acting on this aspect of bone conversion.

*H. pylori* infection can affect nutrient absorption in the digestive system and thus alter the nutritional status of the whole body [26]. Nutritional status can be measured indirectly by the serum albumin level and the body mass index. One study found that the *H. pylori* infection rate was lowest when the serum albumin level was ≥ 48 g/L and highest when the serum albumin was < 45 g/L. A significant correlation between *H. pylori* infection and serum albumin was observed [27]. The present study found that the level of serum albumin in the positive group was 40.80 g/L, which was lower than that in the negative group (47.30 g/L), with a statistically significant difference. This suggests that the presence of *H. pylori* could reduce the serum albumin level, especially when the level was in the region of 40 g/L. A healthy protein intake is very important to bones. Protein can form bone matrix and promote fracture healing and growth [28]. When the protein intake is lower than required for growth, a negative nitrogen balance can delay bone growth in children, although the effects on adult bones remain controversial [29]. It has been reported that bone mineral density decreases in correspondence with reduced albumin levels, although this phenomenon gradually disappeared with age [30]. Here, it was found that the serum albumin level of the normal bone-mass group was higher than that of the low bone-mass and osteoporosis groups and showed a moderate negative correlation in the Spearman test. The incidence of osteoporosis increased as the albumin levels decreased. The logistic regression analysis showed an association between the serum albumin levels and both *H. pylori* infection and osteoporosis, suggesting that *H. pylori* may lead to the development of osteoporosis through abnormal changes in serum albumin. Recent studies have found that albumin and its degradation product, albumin peptides, have an important impact on bone health. Albumin peptides can regulate the balance between bone loss and bone production by interacting with osteoblasts and their surrounding cells [31]. In addition, albumin peptides are able to influence the formation and breakdown of the extracellular matrix, further affecting bone health [31]. When albumin peptide levels are too high or metabolic function is impaired, osteoporosis may result. Obesity is a multifactorial metabolic disorder that promotes biochemical and immunological changes, characterised primarily by expansion of adipose tissue and chronic low-grade systemic inflammation [32]. Adipose tissue from obese patients expresses higher levels of tumour necrosis factor-α (TNF-α), interleukin 6 (IL-6) and C-reactive protein (CRP), and high levels of pro-inflammatory factors are associated with bone loss through activation of nuclear factor (NF)-κB receptor-activating factor ligand (RANKL) [32]. In addition, in obese populations, PPARγ can inhibit osteoblast differentiation through Runx2 reduction, leading to bone loss and osteoporosis [32].

*H. pylori* infection leads to chronic inflammation, the formation of advanced glycation end products, and the production of vasoactive substances such as interleukin and leukotriene, reducing the levels of C-peptide, affecting insulin secretion, increasing insulin resistance, accelerating the decline of islet β-cell function, and finally leading to glucose metabolism disorders, leading to elevated blood sugar that is difficult to control [33]. In the Xu Han study, the prevalence of diabetes after *H. pylori* infection increased significantly from 20.2 to 21.3% [34]. In the present study, FPG in the *H. pylori*-positive group were higher than that in the negative group, which is consistent with most studies [33–34]. *H. pylori* infection can increase FPG level. Although osteoporosis is not included in the complications of diabetes, diabetes increases the risk of osteoporosis [35]. Here, the blood glucose levels of the normal bone-mass group, the low bone-mass group, and the osteoporosis group were 4.99 mmol/L, 4.98 mmol/L, and 5.08 mmol/L, respectively. The blood glucose in the osteoporosis group increased significantly and showed a low positive correlation in the Spearman test. Thus, the incidence of osteoporosis increased with increases in blood glucose. After adjustment for confounding factors, it was found that FPG was associated with both *H. pylori* and osteoporosis, suggesting that *H. pylori* may induce abnormal changes in blood sugar, eventually leading to the development of osteoporosis. When the human body experiences high glucose levels over an extended period, this may affect the early accumulation of bone mass as well as reduce existing bone mass and strength and adversely affect the functions of adjacent bone cells, including endothelial cells, mesenchymal cells, and adipocytes, resulting in osteocytic failure and bone marrow dysfunction [36–37]. Sustained hyperglycaemia induces lipid deposition, poor blood supply, glucose toxicity and oxidative stress that can lead to the development of osteoporosis [38]. Disruption of lipid metabolism causes aggregation of VLDL and TC in the subendothelial and endothelial cell layers leading to atherosclerosis and narrowing of the vascular lumen, resulting in inadequate blood supply to the bone and possible structural abnormalities such as microcracks [38].

A prospective study observed that cholesterol levels in *H. pylori*-positive groups were lower than those in *H. pylori*-negative groups (45.2 mmol/L vs. 47.3 mmol/L). After radical eradication of *H. pylori*, the HDL levels increased from 40.5 mmol/L to 46.3 mmol/L [39]. Kyoichi Adachi reached the same conclusion that the HDL levels in *H. pylori*-positive patients were significantly lower than in uninfected patients (63.9 mmol/L vs. 68.1 mmol/L), and suggested that long-term *H. pylori* infection was related to blood lipid metabolism [40]. In the present study, the HDL level in the *H. pylori*-positive group was lower than that in the negative group (1.17 mmol/L vs. 1.32 mmol/L) while the LDL level was higher in the positive group than in the negative group (2.30 mmol/L vs. 2.18 mmol/L). A study of elderly women in Japan found that the spinal bone mineral density decreased with increased LDL and was reduced as the HDL levels decreased, finding a positive correlation between HDL and bone mineral density in postmenopausal women [41]. However, in an investigation of male osteoporosis patients by Framingham, the forearm bone mineral density decreased slightly with increased cholesterol levels, although there was no significant correlation observed in older women. CHL had no long-term effect on bone mineral density [42]. Here, significant differences in TG, HDL, and LDL levels were observed among the three groups, although there were no significant differences in the cholesterol levels among the three groups. The Spearman correlations showed a positive correlation between LDL and bone mineral density and negative correlations between TG and HDL. The incidence of osteoporosis thus increased with decreased HDL levels. The logistic regression analysis showed that HDL was closely associated with both *H. pylori* infection and osteoporosis, suggesting that *H. pylori* may affect bone mineral density through HDL. HDL-C plays a multifaceted role in many other biological processes, including inflammation, oxidative stress, nitric oxide production, and regulation of plasma glucose homeostasis. HDL-C promotes cholesterol efflux from osteoclasts by up-regulating ABCG1 expression, removes oxysterols from the peripheral circulation, and induces apoptosis in osteoclasts and affects their formation, thereby decreasing the levels of factors associated with bone resorption [43].

The body obtains Vitamin D in essentially two ways, namely, intake from food and nutritional supplements and Vitamin D synthesis in the skin. Vitamin D binds to intestinal, parathyroid, kidney, and bone receptors to regulate the levels of calcium and phosphorus in the plasma, and subsequently regulates osteoblasts and osteoclasts to maintain healthy bone metabolism. Long-term vitamin D deficiency can cause progressive bone loss and lead to osteoporosis [44]. In 2007, a study by the United States Health Care Agency demonstrated a clear correlation between vitamin D and bone mineral density [45]. In the present study, the vitamin D levels in the normal bone-mass group, the low bone-mass group, and the osteoporosis group were 19.88 ng/ml, 13.87 ng/ml, and 12.62 ng/ml, respectively. The differences were statistically significant. The Spearman test showed a low negative correlation. A Japanese study on elderly women found that the incidence of osteoporosis increased when vitamin D levels were reduced and recommended vitamin D supplementation to treat and prevent osteoporosis. The *H. pylori* infection rate was found to be lower than that in people without vitamin D treatment [46]. Vitamin D can not only regulate the metabolism of calcium and phosphorus to affect osteoporosis but can also prevent and treat *H. pylori* infections. Here, it was also found that the vitamin D levels were significantly lower in the *H. pylori*-positive group than in uninfected patients. Logistic regression analysis showed that vitamin D levels were associated with both *H. pylori* infection and osteoporosis, suggesting that vitamin D may mediate the association between *H. pylori* infection and osteoporosis. vitamin D acts indirectly on bone by affecting the immune system and inflammatory processes. VD and its metabolites affect innate immunity by promoting macrophage development and activation, resulting in the production of defensins, including histones and 2-defensins, and the antimicrobial factors IL-6, TNF, and IL-1. VD deficiency activates specific T-cell subsets to produce IL-17, a receptor activator of nuclear factor kappa B ligand (RANKL), IL-1, TNF, and IL-6. IL-1, TNF, and IL-6, which stimulate osteoclast maturation and activity by preventing osteoblast differentiation, increasing osteoclast apoptosis, and increasing RANKL expression and the RANKL/osteoprotegerin (OPG) ratio[47].

However, there are some limitations to this study. First, despite careful adjustment for various confounders in observational analyses, residual and unmeasured confounders may have remained biased in our study. Second, due to limitations of the genetic data used in the GWAS, we were unable to stratify our analyses according to factors of interest (e.g., age or sex). Third, although our findings highlight a causal relationship between *H. pylori* infection and osteoporosis, it relies on a set of pre-existing assumptions, and future clinical studies are needed to confirm causality and explore potential mechanisms.

## Conclusion

Our study provides evidence for causal effects of *H. pylori* infection on osteoporosis. *H. pylori* may affect osteoporosis through serum albumin, high-density lipoprotein, fasting blood glucose, and vitamin D. Thus, in the clinic, infection with *H. pylori* should not be ignored in the management of osteoporosis.

### Electronic supplementary material

Below is the link to the electronic supplementary material.

Supplementary Material 1

Supplementary Material 2

Supplementary Material 3

Supplementary Material 4

## Data Availability

The datasets generated and/or analyzed during the current study are available from the corresponding author on reasonable request.

## Abbreviations

BMI: body mass index
SBP: systolic blood pressure
DBP: diastolic blood pressure
WBC: white blood cell
NEU: neutrophil percentage
LYM: lymphocyte percentage
CA: calcium
P: phosphorus
ALP: alkaline phosphatase
ALB: albumin
FBG: fasting blood glucose
TP: Total protein
SCr: serum creatinine
UA: uric acid
TC: total cholesterol
TG: triglyceride
HDL-C: high-density lipoprotein
LDL-C: low-density lipoprotein
CRP: C-reactive protein
VitD: vitamin D
PTH: parathormone
OC: osteocalcin
β: Standardized partial regression coefcient
CI: Confdence interval
OR: Odds ratio
