# Supplementary material for: H. pylori infection and osteoporosis: a large-scale observational and mendelian randomization study
Source: BMC Infect Dis. 2024 Mar 12;24:305. doi: 10.1186/s12879-024-09196-1 (PMC10935925; doi:10.1186/s12879-024-09196-1)
Supplement: Supplementary file 1 — Supplementary Material 1 [file 12879_2024_9196_MOESM1_ESM.docx]

**Supplementary Table 1** :Details of the studies included in the Mendelian randomization analyses

| Phenotype | Consortium  or author | Ethnicity | Sample size | Year | Number  of SNPs | Web source |
| --- | --- | --- | --- | --- | --- | --- |
| H. polyri infection E | EBI | European | 1,058 cases and 3,625 controls | 2021 | 7,247,045 | https://gwas.mrcieu.ac.uk/datasets/ieu-b-4905/ |
| Osteoporosis | MRC-IEU | European | 5,266 cases and 331,893 controls | 2017 | 10,894,596 | https://gwas.mrcieu.ac.uk/datasets/ukb-a-87/ |
