# Supplementary material for: H. pylori infection and osteoporosis: a large-scale observational and mendelian randomization study
Source: BMC Infect Dis. 2024 Mar 12;24:305. doi: 10.1186/s12879-024-09196-1 (PMC10935925; doi:10.1186/s12879-024-09196-1)
Supplement: Supplementary file 2 — Supplementary Material 2 [file 12879_2024_9196_MOESM2_ESM.docx]

**Supplementary Table 2**: Associations of genetic instruments for *H. pylori* infection with osteoporosis.

| beta.exposure | se.exposure | pval.exposure | id.exposure | SNP | effect_allele.exposure | other_allele.exposure | eaf.exposure |
| --- | --- | --- | --- | --- | --- | --- | --- |
| 0.315775 | 0.0672338 | 2.74183E-06 | ieu-b-4905 | rs41263973 | A | G | 0.033534 |
| -0.105911 | 0.0230674 | 4.55229E-06 | ieu-b-4905 | rs2169557 | T | C | 0.48846 |
| 0.18787 | 0.0401253 | 2.94307E-06 | ieu-b-4905 | rs77516628 | T | A | 0.088953 |
| -0.229692 | 0.0481386 | 1.90042E-06 | ieu-b-4905 | rs72708546 | A | G | 0.059115 |
| -0.175163 | 0.0342852 | 3.40432E-07 | ieu-b-4905 | rs35030589 | A | G | 0.13205 |
| 0.405025 | 0.0865994 | 3.01655E-06 | ieu-b-4905 | rs117912702 | A | G | 0.019949 |
| 0.212576 | 0.0440791 | 1.47479E-06 | ieu-b-4905 | rs73512476 | T | G | 0.076735 |
| -0.396711 | 0.0846646 | 2.89208E-06 | ieu-b-4905 | rs17502937 | T | G | 0.020036 |
| -0.17464 | 0.038012 | 4.48818E-06 | ieu-b-4905 | rs74045808 | T | C | 0.10756 |
| 0.31781 | 0.0685235 | 3.64158E-06 | ieu-b-4905 | rs78825412 | A | C | 0.032426 |
| -0.128595 | 0.0265143 | 1.28591E-06 | ieu-b-4905 | rs12591869 | A | C | 0.26981 |
| 0.29919 | 0.0647964 | 4.01976E-06 | ieu-b-4905 | rs55871438 | C | T | 0.039419 |

***P* value < 5×10^-8^ for reporting genome-wide significance; SNP, single nucleotide polymorphism.**
