# Supplementary material for: H. pylori infection and osteoporosis: a large-scale observational and mendelian randomization study
Source: BMC Infect Dis. 2024 Mar 12;24:305. doi: 10.1186/s12879-024-09196-1 (PMC10935925; doi:10.1186/s12879-024-09196-1)
Supplement: Supplementary file 3 — Supplementary Material 3 [file 12879_2024_9196_MOESM3_ESM.docx]

**Supplementary Table 3**: Associations of genetic instruments for osteoporosis with *H. pylori* infection.

| se.exposure | beta.exposure | pval.exposure | id.exposure | SNP | effect_allele.exposure | other_allele.exposure | eaf.exposure |
| --- | --- | --- | --- | --- | --- | --- | --- |
| 0.000395973 | -0.00211508 | 9.22529E-08 | ukb-a-87 | rs6684375 | T | C | 0.175063 |
| 0.000381881 | -0.00191554 | 5.27667E-07 | ukb-a-87 | rs1414660 | T | C | 0.192011 |
| 0.000360894 | -0.00165414 | 4.57509E-06 | ukb-a-87 | rs10931982 | C | T | 0.774507 |
| 0.000348924 | -0.00160268 | 4.36657E-06 | ukb-a-87 | rs10194857 | C | T | 0.246783 |
| 0.000449257 | 0.00226354 | 4.69645E-07 | ukb-a-87 | rs28402693 | T | C | 0.130168 |
| 0.000302262 | -0.00191327 | 2.45731E-10 | ukb-a-87 | rs10490823 | T | C | 0.556175 |
| 0.000322204 | 0.00150091 | 3.19029E-06 | ukb-a-87 | rs6843623 | C | A | 0.33212 |
| 0.000318431 | 0.00206293 | 9.28111E-11 | ukb-a-87 | rs7683315 | A | T | 0.66265 |
| 0.00112624 | 0.00536473 | 1.90423E-06 | ukb-a-87 | rs34932159 | T | G | 0.0182874 |
| 0.000336089 | -0.00249722 | 1.08668E-13 | ukb-a-87 | rs3801387 | G | A | 0.278173 |
| 0.000302704 | -0.00199254 | 4.6334E-11 | ukb-a-87 | rs10280461 | A | C | 0.545389 |
| 0.000320923 | 0.00176493 | 3.81092E-08 | ukb-a-87 | rs1548607 | G | A | 0.326982 |
| 0.000578604 | 0.00301364 | 1.90507E-07 | ukb-a-87 | rs74777717 | G | C | 0.072905 |
| 0.000354576 | 0.00169808 | 1.67656E-06 | ukb-a-87 | rs3094751 | A | G | 0.234774 |
| 0.000302094 | 0.00151323 | 5.46978E-07 | ukb-a-87 | rs2450083 | C | T | 0.497107 |
| 0.00153214 | 0.00714798 | 3.08198E-06 | ukb-a-87 | rs62551986 | C | A | 0.0103248 |
| 0.000730723 | 0.00359575 | 8.62263E-07 | ukb-a-87 | rs79751840 | C | T | 0.0443803 |
| 0.000336196 | 0.00229604 | 8.5369E-12 | ukb-a-87 | rs11228240 | T | C | 0.278773 |
| 0.000301161 | 0.00140206 | 3.23311E-06 | ukb-a-87 | rs11231740 | T | C | 0.479699 |
| 0.000306966 | 0.00158514 | 2.42019E-07 | ukb-a-87 | rs4542364 | A | G | 0.412037 |
| 0.000331372 | 0.00180259 | 5.33777E-08 | ukb-a-87 | rs7303922 | G | A | 0.707214 |
| 0.000300962 | 0.00205707 | 8.21486E-12 | ukb-a-87 | rs9594738 | T | C | 0.489103 |
| 0.000744548 | 0.00344258 | 3.77042E-06 | ukb-a-87 | rs12588965 | A | T | 0.0455133 |
| 0.000343784 | 0.0016537 | 1.50782E-06 | ukb-a-87 | rs3742909 | A | G | 0.260631 |
| 0.000958479 | 0.00438493 | 4.76661E-06 | ukb-a-87 | rs142809063 | T | C | 0.0270078 |
| 0.000779808 | 0.00361092 | 3.64872E-06 | ukb-a-87 | rs71378512 | A | G | 0.0451366 |
| 0.000565915 | -0.0028275 | 5.84602E-07 | ukb-a-87 | rs188810925 | A | G | 0.0803324 |
| 0.00030249 | -0.00143207 | 2.19933E-06 | ukb-a-87 | rs9636107 | G | A | 0.467716 |
| 0.000468789 | 0.0022558 | 1.49517E-06 | ukb-a-87 | rs34325313 | C | A | 0.118512 |

***P value < 5×10-8 for reporting genome-wide significance; SNP, single nucleotide polymorphism.***
