# Supplementary figures and images for: H. pylori infection and osteoporosis: a large-scale observational and mendelian randomization study
Source: BMC Infect Dis. 2024 Mar 12;24:305. doi: 10.1186/s12879-024-09196-1 (PMC10935925; doi:10.1186/s12879-024-09196-1)

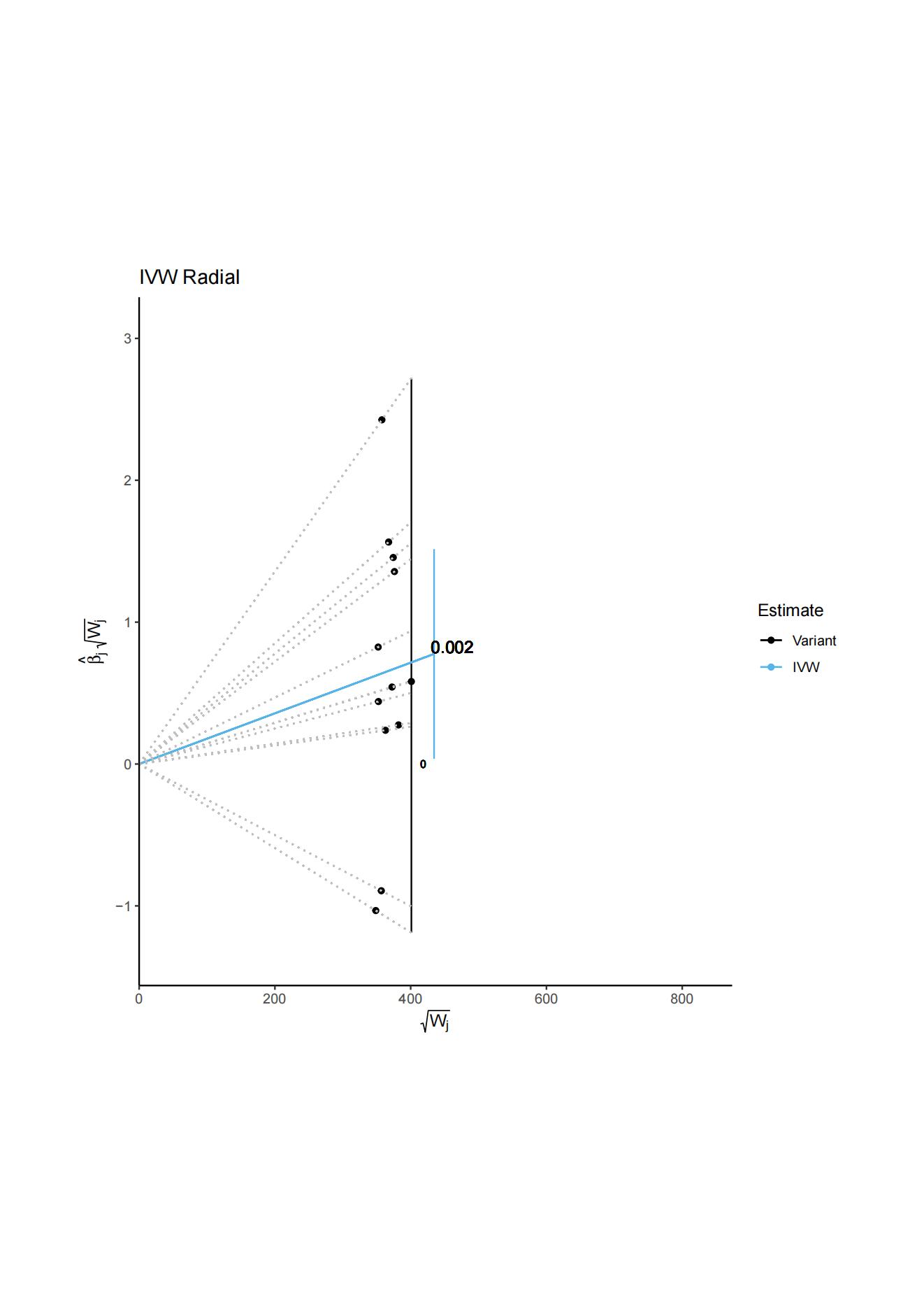

Supplement: Supplementary file 4 — Supplementary Material 4 [file 12879_2024_9196_MOESM4_ESM.jpg]
